# Microfibrillated Cellulose Suspension and Its Electrorheology

**DOI:** 10.3390/polym11122119

**Published:** 2019-12-17

**Authors:** Kisuk Choi, Jae Do Nam, Seung Hyuk Kwon, Hyoung Jin Choi, Md Sakinul Islam, Nhol Kao

**Affiliations:** 1 Department of Polymer Science and Engineering, Sungkyunkwan University, Suwon 16419, Korea; kisuk929@skku.edu (K.C.); jdnam@skku.edu (J.D.N.); 2 Department of Polymer Science and Engineering, Inha University, Incheon 22212, Korea; focalis@naver.com; 3 School of Engineering, Chemical and Environmental Engineering, RMIT University, Melbourne, Victoria 3000, Australia; s3390737@student.rmit.edu.au (M.S.I.); nhol.kao@rmit.edu.au (N.K.)

**Keywords:** microfibrillated cellulose, rice husk, electrorheological fluids, suspension

## Abstract

Microfibrillated cellulose (MFC) particles were synthesized by a low-pressure alkaline delignification process, and their shape and chemical structure were investigated by SEM and Fourier transformation infrared spectroscopy, respectively. As a novel electrorheological (ER) material, the MFC particulate sample was suspended in insulating oil to fabricate an ER fluid. Its rheological properties—steady shear stress, shear viscosity, yield stress, and dynamic moduli—under electric field strength were characterized by a rotational rheometer. The MFC-based ER fluid demonstrated typical ER characteristics, in which the shear stresses followed the Cho–Choi–Jhon model well under electric field strength. In addition, the solid-like behavior of the ER fluid was investigated with the Schwarzl equation. The elevated value of both dynamic and elastic yield stresses at applied electric field strengths was well described using a power law model (~E^1.5^). The reversible and quick response of the ER fluid was also illustrated through the on–off test.

## 1. Introduction

Smart bio-based fibers and polymers, originated from biomass feedstock and renewable agriculture, have attracted a huge amount of attention recently due to their abundant source and ascending concern of the environmental issue of petroleum-based polymers [1]. Among these, cellulose is one of the most ample polymers, with its annual production being about 1.5 × 10^12^ tons [2]. Many studies have proven that natural fibers or cellulosic fibers could become an alternative mineral/inorganic reinforcing fiber in composites [3,4,5,6]. Whilst natural fibers consist of many benefits—such as low density, biodegradability, abundance, and renewability—pristine lignocellulosic fillers are constrained in industrial usage because of their insufficient mechanical properties, even though they possess good specific mechanical properties at low density [3].

In general, among various cellulosic fibers, the microfibrillated cellulose (MFC) is the one fabricated by the delamination of cellulose fibers using a high-pressure homogenizer [7,8]. The mechanical shear force induced by a high-pressure homogenizer propagates to fibrillation of the cellulosic fibers, resulting in microfibrils with the diameter of 10–100 nm and a web-like form. The fabrication of MFC does not require acid hydrolysis, which would be the next step required for converting microfibrils to cellulose nanowhiskers or cellulose nanofibrils. This process is not only time consuming and expensive, due to the filtration, but it also requires a dialysis process to remove the remaining acid. Other processes include the use of a grinder or a microfluidizer for mechanical fibrillation [9]. For instance, cotton, tunicin, wood pulp, and bacterial celluloses can be broken down to nanofibers with the oxidation of 2,2,6,6-tetramethylpiperidine-1-oxyl (TEMPO) radical and mechanical treatment [10]. A mild enzymatic hydrolysis has also drawn increasing attention. It enables the ability to control the delamination of long/entangled cellulose when combined with the high-pressure homogenization and mechanical shearing [11]. New sources, new mechanical processes, and new pre- and post-treatments are being continuously developed to reduce the high energy consumption used to fabricate a new type of MFC particles for engineering applications [12]. From this point of view, MFC has great potential to be used as a bio-based structural-reinforcing cost-efficient material [13]. Therefore, a good understanding of the rheological and other properties of the MFC is required.

Concurrently, electroresponsive smart and intelligent electrorheological (ER) fluids, which comprise of dielectric dispersoid particles in an insulating liquid, exhibit a chain-like form under a strong applied electric field due to the dielectric constant disproportion of the suspended particles [14]. The sudden phase change of polarized dispersed particles, of ER fluids from solid-like to liquid-like under a strong applied direct current (DC) electric field, makes their mechanical and viscoelastic behaviors controllable by an applied electric field. Therefore, their corresponding rheological properties have been investigated by various range of rheological equations of state with a yield stress, including the Seo–Seo [15], Herschel–Bulkley [14], and Cho–Choi–Jhon [16] models. Most of the actively responsive particles adopted in ER fluids are known to be polarizable particles, including dielectric inorganics (such as SiO_2_, TiO_2_, and BaTiO_3_), or conducting polymers with appropriate conductivity in between 10^−6^ and 10^−9^ S/m. These particles can be easily polarized to form a fibrillated structure in the direction of the applied electrical field with their changeable rheological properties [17]. It can be noted that the ER systems are generally classified into two categories—that is, hydrous or anhydrous ER systems—and the carrier species for particle polarization differentiates the classification between wet and dry ER systems [18]. The existence of branched polar groups or conducting repeating units such as amine, hydroxyl, and amino-cyano are required for the ER functionality of micron-sized particles. Polar groups affect ER behavior under an applied voltage by behaving as an electron donor [14]. On the other hand, cellulose has been applied as a water-based ER fluid because of the presence of a small trace of water [19]. Therefore, the cellulose particles in addition to chitosan, corn starch, and others have been adopted as biopolymeric particles for ER suspensions [20,21].

Recently, microcrystalline cellulose (MCC) synthesized from rice husk in a three-stage procedure of alkali treatment, bleaching, and hydrolysis was introduced as ER particles [19], while Davies et al. [22] examined the ER behavior of MCC in various oils, and Yatsuzuka et al. [23] reported an ER behavior of rod-like commercial MCC. However, the MFC has never been adopted as the ER material before, even though studies on MFC systems have been extensively conducted revealing their promising and interesting properties, which may be suitable for various industrial fields and products usage [24,25,26,27,28,29]. For this reason, MFC was selected as a dispersed phase of the ER suspension in this study. Its rheological behaviors at various electrical strengths are presented and discussed in this paper.

## 2. Materials and Methods 

### 2.1. Materials and Fabrication of MFC

Pristine rice husk biomass powder was used to produce MFC through a low-pressure alkaline delignification procedure, which has already been reported in the literature [30]. The raw rice husk biomass powder, whose particle size in the range of 100–700 µm, was used for delignification. The delignification was performed at 70 °C with 2.5 M NaOH solution for 9 h (Batch No. LPR-06). Delignification was mainly carried out to separate non-cellulosic parts such as lignin, hemi-cellulose, silica, and waxy materials, etc. from the rice husk. At the end of delignification, the delignified rice husk pulp was separated from the black liquor by centrifugation and then washed five times with deionized water. Finally, the washed delignified rice husk pulp was neutralized by 20% dilute sulfuric acid and again cleaned five times to remove sulfate salts from delignified pulp. Then, it was dried in an oven at 45 °C for 72 h to reach constant weight. The drying was performed at low temperature to prevent cellulose pulp from thermal degradation. Then, bleaching was carried out on the dried delignified rice husk pulp by 20% sodium hypochlorite solution at 60 °C for 60 min under continuous magnetic stirring at 800 rpm. The bleached rice husk pulp was isolated from reaction solution through centrifugation, then washed and neutralized in the same method used for the delignified sample. Finally, the bleached sample was dried in an oven until it reached the constant mass. The dried bleached rice husk pulp contained (85%–90%) cellulose content and the balance consisted of non-cellulosic components (10%–15%). This dried bleached rice husk pulp was converted to powder form by grinding and stored for further analyses and uses. This ground bleached rice husk pulp is known as MFC, which was confirmed by a morphological analysis. The compositional analysis was performed by the Technical Association of the Pulp and Paper Industry (TAPPI) method for both delignified and bleached rice husk pulps.

### 2.2. Fabrication of MFC-Based ER Fluid

The MFC-based ER suspension in this study was synthesized by suspending the MFC particle (10 vol%) in an insulating silicone oil (100 cS). Then, the suspension was mechanically stirred and ultrasonicated to enhance its uniform dispersion.

### 2.3. Characterization

Both microstructure and surface morphology were observed by TESCAN Vega scanning electron microscopy (SEM) (Tescan USA Inc., Warrendale, PA, USA). This SEM test was performed at the RMIT Microscopy and Microanalysis Facility (RMMF). For the morphological test, the samples were loaded on aluminum stubs with conducting carbon tape without any surface treatment. The Fourier transformation infrared (FT-IR) spectra of MFC particles were obtained using an FT-IR spectrometer (Spectrum 100, Perkin-Elmer, Waltham, MA, USA) at an ambient condition at RMIT. The transmittance mode of the spectra was collected from 20 accumulated scans at a 4 cm^−1^ resolution over a 4000–400 cm^−1^ window. On the other hand, the viscoelastic characteristics of the shear stress, shear viscosity, and dynamic modulus values of the ER suspension at different electrical fields were characterized using a rotational rheometer (MCR 300, Anton Paar, Graz, Austria) with a high-voltage generator and concentric cylinder geometry (CC 17/E).

## 3. Results and Discussion

**Figure 1:**
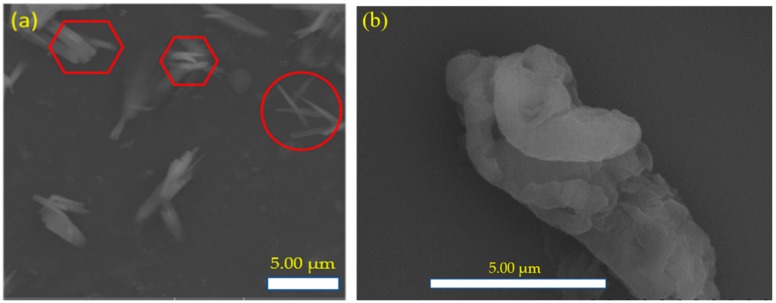
SEM images of microfibrillated cellulose (MFC) with at low (**a**) and high (**b**) magnification.

The SEM images of MFC are presented in Figure 1a,b, which were captured at low and high magnification with the same spatial resolution. However, these two images were captured for the same specimens focusing on two different areas to investigate the MFC particles. MFC particles were rod-like shaped, in which their width was in the range of 500–1000 nm and length in several micrometers less than 10 µm. In both images, the individual MFC particles and their agglomeration are shown by the circle and hexagon, respectively. The dimensions of MFC as observed from SEM in Figure 1 were similar to those previously reported [30,31,32,33].

**Figure 2:**
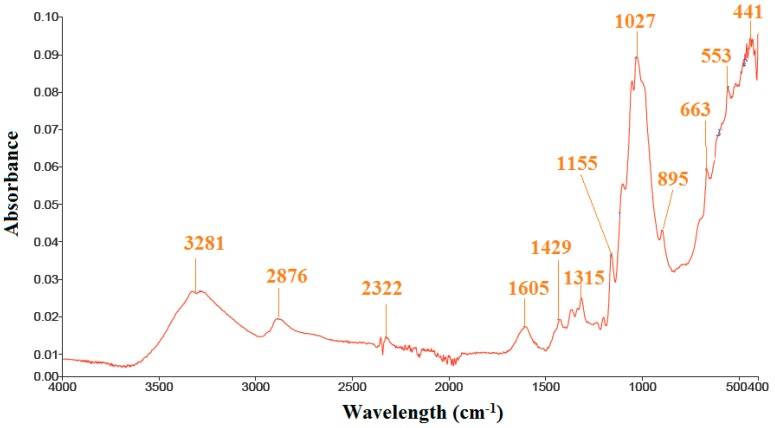
FT-IR spectra of MFC.

Significant numbers of FT-IR peaks for band position and possible stretching were observed for MFC as given in Figure 2 and Table 1. The appearance of a peak at 3281 cm^−1^ was associated with the –OH bending vibration, while the peak at 2876 cm^−1^ was probably due to the C–H stretching of MFC. The bending vibrations of C–O and C–H observed at 800–1400 cm^−1^ were due to the cellulose structure of the MFC. There was a weak peak at 2322 cm^−1^, which was possibly due to alkaline stretching, but this is unreliable in the case of MFC. Moreover, the peaks at 1605 cm^−1^ were due to the removal of lignin in the MFC. Furthermore, the peaks near 450 cm^−1^ and 800 cm^−1^ seemed to be due to the silica of MFC apart from raw rice husk. The FT-IR results of MFC in this study were in good agreement with other published works [34,35,36,37].

**Figure 3:**
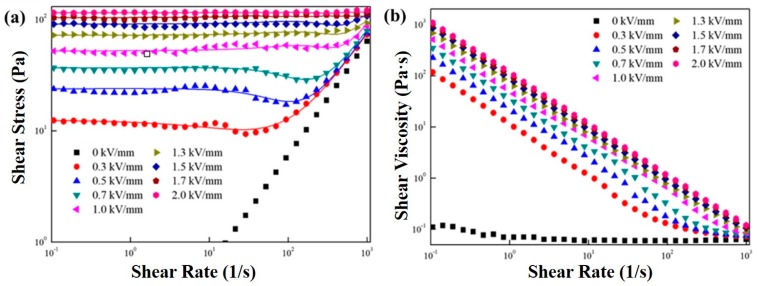
Flow curve of shear stress (**a**) and shear viscosity (**b**) of the MFC-based electrorheological (ER) fluid under various electric filed strengths. The line in (**a**) is fitted by the Cho–Choi–Jhon (CCJ) model.

Figure 3 shows the flow curves of MFC-based ER fluid measured from a controlled shear rate (CSR) method of the rheological measurement under a shear rate window from 0.1 to 1000 s^−1^. Its shear stress increased as a function of an applied shear rate without an electrical field strength following a Newtonian fluid property. As soon as an electrical field was introduced, the MFC-based ER suspension immediately showed a non-Newtonian fluid property, e.g., demonstrating a yield stress at a low shear rate limit, and the shear stress increased with the increased electric field strength. In a low shear rate window, the plateau region of shear stresses was being widened along with increased electrical field strength of up to 2.0 kV/mm. This plateau behavior is considered to be originated by an attractive force by the polarized particles, building a chain structure. Here, the Cho–Choi–Jhon (CCJ) equation was used to fit the shear stress curves. The CCJ model in Equation (1) [38] is known to be a proper rheological equation of state to describe the flow curves of various ER fluids for a wide shear rate region.

(1) $$\tau =\frac{\tau_{y}}{1+{\left(t_{2}\dot{\gamma }\right)}^{\alpha }}+\eta_{\infty }\left(1+\frac{1}{{\left(t_{3}\dot{\gamma }\right)}^{\beta }}\right)\dot{\gamma }$$

where τ_y_ is the yield stress obtained from a shear stress at a zero shear rate extrapolation, t_2_ and t_3_ are the time constants, and η_∞_ is the high shear viscosity. Although α is designated to the shear stress decrease at a low shear rate regime, the constant β is located in a range of 0 < β ≤ 1, because dτ/dγ ≥ 0 [39].

Figure 3b presents the shear viscosity as a function of the shear rate at various electrical field strengths. The MFC particle-based ER fluid showed a Newtonian fluid behavior for the wide shear rate window at the zero-electric field. The shear viscosity of MFC particle-based ER suspension dramatically increased with the increasing electrical field strength due to the build-up of a chain structure in the presence of electrical field strength. In addition, the increment of shear stress and shear viscosity was due to the interfacial polarization of dispersed ER particles. The interfacial polarization under an external electric field causes electrostatic forces between particles, leading to the chain structural formation [19]. In addition, the shear thinning behavior was observed for the wide shear rate regime. This indicated that the initially formed chain structure of the MFC sample was deformed and then broken by the hydrodynamic shear flow, but still keeping some solid-like structures along with the streamline.

**Figure 4:**
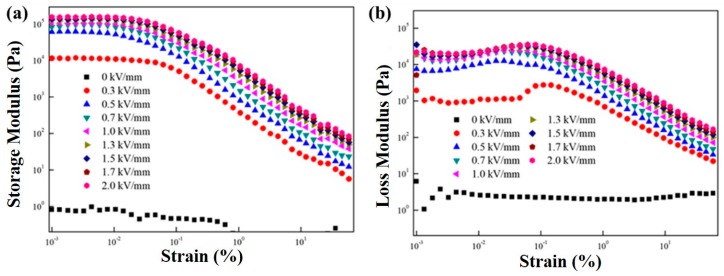
The storage modulus (**a**) and loss modulus (**b**) of the MFC-based ER fluid as a function of strain under various electric field strengths.

A dynamic oscillation measurement of the MFC particle-based ER suspension was performed to estimate the linear viscoelastic (LVE) regime in the strain amplitude sweep study at a constant angular frequency of 1 Hz in the strain region from 0.001% to 100%, as shown in Figure 4. The loss moduli were slightly higher than the storage moduli, indicating that the MFC particle-based ER suspension possessed a liquid-like property without electrical field strength. However, the storage moduli became higher than the loss moduli as electrical field strength was applied, indicating that the phase characteristics of the ER suspension moved from fluid-like to solid-like by the electrical field strength. At the low strain region (LVE region, <0.005%), a plateau storage modulus regime was found, because the structural deformation and reformation was reversible. Above the LVE region (>0.005%), both storage (G′) and loss (G″) moduli decreased due to the irreversible structural change. Consequently, a 0.005% strain value was selected for the angular frequency sweep test.

**Figure 5:**
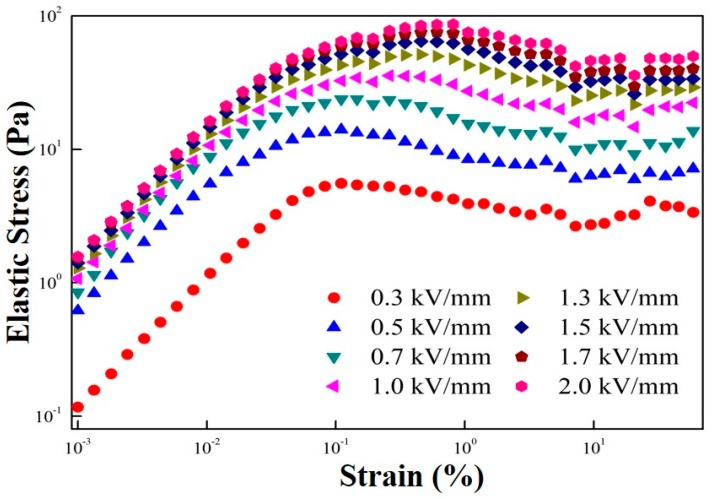
Elastic stresses of the MFC-based ER fluid under various electric field strengths.

Figure 5 shows the elastic stresses of the ER suspension for different electrical field strengths. This elastic stress based on the strain amplitude sweep study was used to deduce the elastic yield stress, which is different from both static and dynamic yield stresses estimated from a steady shear test. As shown in Figure 5, at a low strain region, the elastic stresses increased almost linearly with the shear strain, implying that the ER suspension responded to the deformation elastically. Around the critical strain value, which is related to the deflection point, their slopes changed. The maximum value of the elastic stress showed a quantitative way of identifying the yield point.

**Figure 6:**
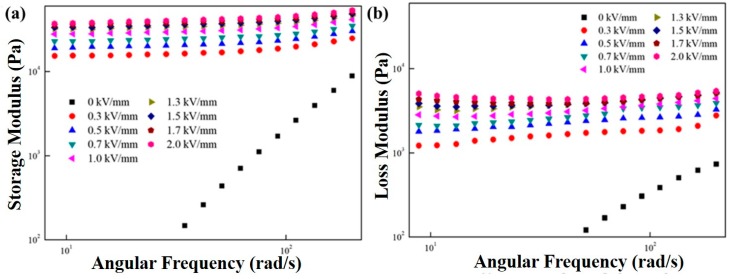
The storage modulus (**a**) and loss modulus (**b**) of the MFC-based ER fluid as a function of angular frequency at fixed strain under various electric field strengths.

Figure 6 represents the angular frequency sweep test result of the MFC-based ER suspension at a constant strain value (γ_LVE_ = 0.005%) over an angular frequency region from 1 to 200 rad/s under different electric field strengths. As presented in Figure 6, the storage and loss moduli showed a fluid-like property that increased with increasing angular frequency without an electric field input. With the input of electrical field strength, the storage modus values were higher than those of the loss modulus, demonstrating that the solid-like characteristic with a stable plateau region prevailed over the viscous behavior. In addition, constant storage modulus values indicated that the fibrillar structure of the ER suspension was not broken in this window of angular frequency.

**Figure 7:**
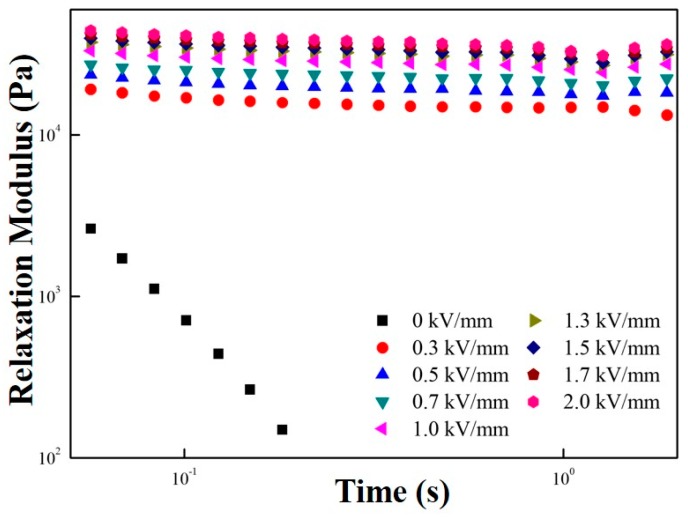
Relaxation modulus of the MFC-based ER fluid.

The solid-like property of the ER suspension could also be explained using Equation (2), which is called the Schwarzl equation as given in Figure 7 [40]. The G′, G″, and ω were obtained from the data in Figure 6. The Schwarzl equation was used to prove the change of fluid behaviors from a fluid-like to a solid-like phase. The Schwarzl equation is expressed as follows:

G(t) ≅ G′(ω) − 0.566G″(ω/2) + 0.203G″(ω).(2)

The relaxation modulus (G(t)) in Equation (2) describes the time-dependent relaxation characteristic of the MFC particle-based ER suspension. From Equation (2), a short-time relaxation behavior of the material can be predicted. At the zero-electrical field strength, the relaxation modulus of MFC particle-based ER fluid decreased sharply and showed a fluid-like behavior. However, when the electrical field strengths were applied, the plateau window appeared, showing its solid-like property.

**Figure 8:**
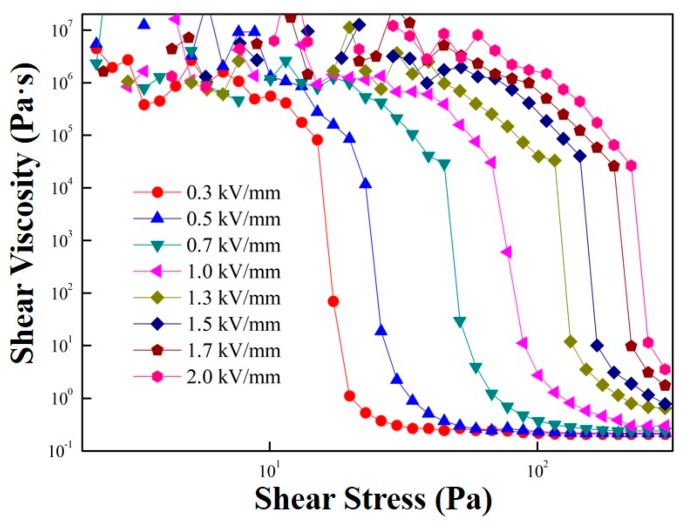
Shear viscosity versus shear stress measured in the controlled shear stress (CSS) mode for MFC particle-based ER fluid under various electric field strengths.

**Figure 9:**
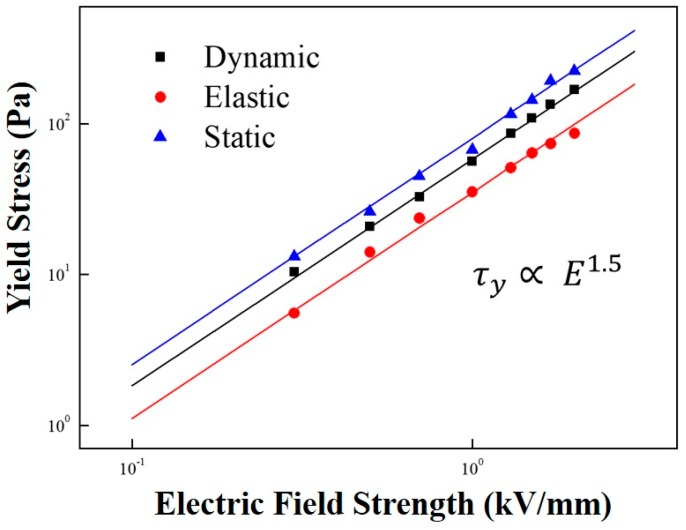
Yield stress dependency on the electric field strength of the MFC-based ER fluid.

As shown in Figure 8 and Figure 9, the shear viscosity change is plotted as a function of an input shear stress. At a low shear stress range, the shear viscosity remained constant but decreased sharply at a critical shear stress value. The sharp decrease in the shear viscosity at this shear stress is regarded as a static yield stress.

Figure 9 gives the correlation between yield stress and electrical field strength. Note that the dynamic yield stress was estimated from the CSR measurement (Figure 3a), while the elastic yield stress was obtained from an amplitude sweep test (Figure 5). Finally, the static yield stress was extracted from the controlled shear stress test (Figure 8). Generally, the relation between the electric field strength and yield stress follows a power-law model (Equation (3)) [41].

(3) $$\tau_{y}\propto E^{m}$$

Similar values of the dynamic, elastic, and static yield stresses are presented in Figure 9; all show the same slope of approximately 1.5 following the conduction mechanism. The slope of the ER fluid less than 2.0 was reported to be related to the indication of insufficient dipole moments from irregular sizes and shapes in the case of the MCC [42,43]. For various ER materials, the elastic yield stress measured from a dynamic test is generally reported to be slightly lower than that determined from the steady-state test. In addition, the static yield stress was slightly higher than the dynamic yield stress obtained from the controlled shear rate test.

**Figure 10:**
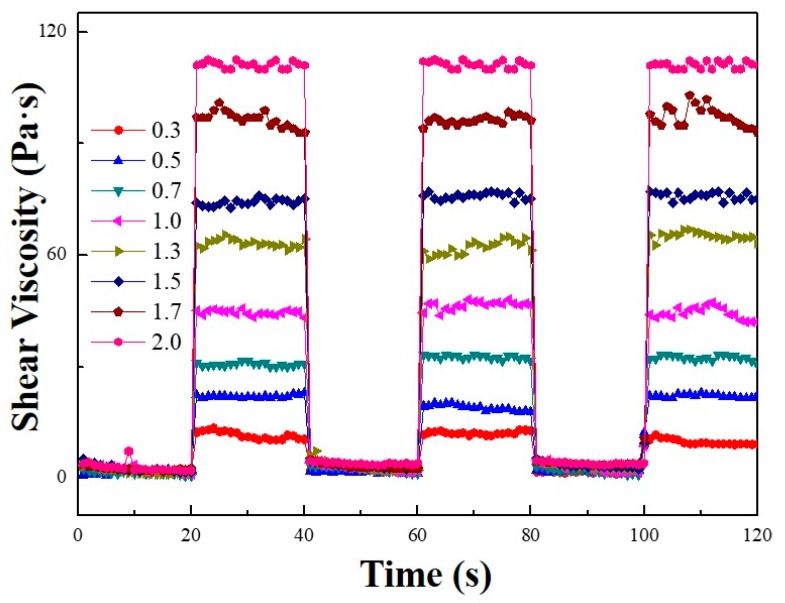
On–off test results of the MFC-based ER fluid at a constant shear rate (= 1 s^−1^) under various electric field strengths with the unit of kV/mm.

Figure 10 illustrates the electrical field “on–off” test on the shear viscosity behavior, which was measured at a fixed shear rate (=1 s^−1^) and square voltage pulse (20 s), at various voltages (0.3–2.0 kV/mm). The results indicated the sensitivity and reliability of the MFC-based ER suspension. For each electric field applied, the shear stress grew quickly to a certain high level when the electrical field was applied and dropped to zero when the electrical field was turned off. At each turning point, the shear stress change of the ER fluid was rapid without any hysteresis, implying a reversible and fast change of the chain-like structure in the ER fluid under an electrical field.

## 4. Conclusions

In this study, the ER test analysis of MFC particles from rice husk was conducted. The behavior of the ER fluid of MFC dispersoids in silicone medium oil was investigated under various applied electrical field. The removal of lignin and silica from rice husk was confirmed in FT-IR and TAPPI analyses of the delignified rice husk pulp, which proved that MFC was well synthesized. SEM image analysis confirmed the rod-like morphology of the MFC particles. The viscoelastic behaviors of the MFC-based ER fluid were investigated using a rotational rheometer. The chain-like structure was apparent when an electrical field was applied. The individual flow curves of ER fluid were well explained by the Schwarzl equation, and both elastic and dynamic yield stresses were analyzed with an external electric field, ~E^1.5^. The enhanced reversibility and sensitivity of the MFC-based ER fluid were also described by the on–off test. Accordingly, the MFC particles can be adopted as dispersoids for ER fluids. 

## Figures and Tables

**Table 1:** Assignments of main peaks identified in FT-IR analysis.

Wave Number (cm^−1^)	Assignment
450	Silica
800	Silica
800–1400	C–O and C–H from cellulose
2322	Alkaline stretching
2876	C–H stretching of MFC
3281	–OH bending vibration
